# Clean Low-Biomass Procedures and Their Application to Ancient Ice Core Microorganisms

**DOI:** 10.3389/fmicb.2018.01094

**Published:** 2018-05-25

**Authors:** Zhi-Ping Zhong, Natalie E. Solonenko, Maria C. Gazitúa, Donald V. Kenny, Ellen Mosley-Thompson, Virginia I. Rich, James L. Van Etten, Lonnie G. Thompson, Matthew B. Sullivan

**Affiliations:** ^1^Byrd Polar and Climate Research Center, The Ohio State University, Columbus, OH, United States; ^2^Department of Microbiology, The Ohio State University, Columbus, OH, United States; ^3^Department of Geography, The Ohio State University, Columbus, OH, United States; ^4^Department of Soil, Water and Environmental Science, The University of Arizona, Tucson, AZ, United States; ^5^Department of Plant Pathology and Nebraska Center for Virology, University of Nebraska–Lincoln, Lincoln, NE, United States; ^6^School of Earth Sciences, The Ohio State University, Columbus, OH, United States; ^7^Department of Civil, Environmental and Geodetic Engineering, The Ohio State University, Columbus, OH, United States

**Keywords:** clean, low biomass, *in silico* decontamination, glacier ice, microbial community

## Abstract

Microorganisms in glacier ice provide tens to hundreds of thousands of years archive for a changing climate and microbial responses to it. Analyzing ancient ice is impeded by technical issues, including limited ice, low biomass, and contamination. While many approaches have been evaluated and advanced to remove contaminants on ice core surfaces, few studies leverage modern sequencing to establish *in silico* decontamination protocols for glacier ice. Here we sought to apply such “clean” sampling techniques with *in silico* decontamination approaches used elsewhere to investigate microorganisms archived in ice at ∼41 (D41, ∼20,000 years) and ∼49 m (D49, ∼30,000 years) depth in an ice core (GS3) from the summit of the Guliya ice cap in the northwestern Tibetan Plateau. Four “background” controls were established – a co-processed sterile water artificial ice core, two air samples collected from the ice processing laboratories, and a blank, sterile water sample – and used to assess contaminant microbial diversity and abundances. Amplicon sequencing revealed 29 microbial genera in these controls, but quantitative PCR showed that the controls contained about 50–100-times less 16S DNA than the glacial ice samples. As in prior work, we interpreted these low-abundance taxa in controls as “contaminants” and proportionally removed them *in silico* from the GS3 ice amplicon data. Because of the low biomass in the controls, we also compared prokaryotic 16S DNA amplicons from pre-amplified (by re-conditioning PCR) and standard amplicon sequencing, and found the resulting microbial profiles to be repeatable and nearly identical. Ecologically, the contaminant-controlled ice microbial profiles revealed significantly different microorganisms across the two depths in the GS3 ice core, which is consistent with changing climate, as reported for other glacier ice samples. Many GS3 ice core genera, including *Methylobacterium*, *Sphingomonas*, *Flavobacterium*, *Janthinobacterium*, *Polaromonas*, and *Rhodobacter*, were also abundant in previously studied ice cores, which suggests wide distribution across glacier environments. Together these findings help further establish “clean” procedures for studying low-biomass ice microbial communities and contribute to a baseline understanding of microorganisms archived in glacier ice.

## Introduction

The cryosphere covers approximately 20% of the Earth’s surface, and includes glaciers, snow, ice sheets, permafrost, lake ice, river ice, and sea ice (Fountain et al., 2012). Although microorganisms have been known to be present in glacier ice for nearly a century (McLean, 1919; Darling and Siple, 1941), such early findings were largely ignored until microorganisms were investigated in the deep Vostok ice core in the 1980s (Abyzov et al., 1982; Abyzov, 1993). This motivated further studies of microorganisms in ice cores collected from polar glaciers, such as the Greenland and Antarctic ice sheets (Priscu et al., 1998; Karl et al., 1999; Miteva et al., 2004; Tung et al., 2005; Knowlton et al., 2013), as well as some low-latitude ice caps, such as Guliya, Geladangdong, Zuoqiupu, and Noijinkangsang in China (Christner et al., 2000; Liu et al., 2016), Pastoruri in Peru (Gonzalez-Toril et al., 2015), Sajama in Bolivia (Christner et al., 2000), and Mount Humboldt in Venezuela (Ball et al., 2014).

These studies explored the mechanisms by which microorganisms could be archived in glacier ice, and used culture-dependent and -independent methods to reveal which microorganisms were archived. Microbial cells are buried and archived in glacier ice by three major processes: (i) emission from various sources (e.g., vegetation, soils, water, and rocks) and transportation in the air over the ice sheet by atmosphere currents; (ii) deposition onto the glacier ice surface; and (iii) gradual incorporation into the deeper ice layers as snow accumulates continuously (Santibanez-Avila, 2016) during the post-depositional period. Thus, microorganisms immured in ice cores represent those in the atmosphere at the time of deposition and hence reflect environmental conditions during the same time period (Priscu et al., 2007; Xiang et al., 2009). Previous investigations of the microbial community in polar glaciers (e.g., Miteva et al., 2009, 2015; Santibanez-Avila, 2016) and low-latitude glaciers (e.g., Yao et al., 2008; Chen et al., 2016) have suggested that microbial diversity and abundance preserved in deep ice cores are correlated with dust particle concentrations, local climate conditions, and global atmospheric circulation. Usually the biomass is very low in most glacier ice samples, with the estimated number of microbial cells ranging from 10^2^ to 10^4^ cells ml^-1^ (Miteva, 2008). Bacterial strains have often been recovered and isolated from glacier ice (Christner et al., 2000; Miteva et al., 2004; D’Elia et al., 2008; Zhang et al., 2008). Most of these isolated bacteria were psychrotolerant (D’Elia et al., 2008), which had optimal growth temperatures well above freezing and could be preserved under cold environments such as glacier ice for a long time (Willerslev et al., 2004). A growing number of studies have demonstrated the possibility for *in situ* microbial activity in glacier ice. The concentration of methane at several depths in the lowest 90 m is up to an order of magnitude higher than that at other depths in a 3,053-m-deep Greenland Ice Sheet Project 2 ice core (Tung et al., 2005). The excess methane at those depths was produced via *in situ* metabolism of the methanogenic archaea, which expended their metabolic energy to mainly repair damaged DNA and amino acids rather than for growth (Tung et al., 2005). Iron-reducing bacteria were reported to account for producing most of the excess CO_2_ by reducing Fe^3+^ to Fe^2+^ and oxidizing the organic acids ions to CO_2_ in ice at some depths of the bottom 13 m of the Greenland Ice Sheet Project 2 ice core (Tung et al., 2006). Some dominant genera (e.g., *Acinetobacter*, *Sphingomonas*, and *Comamonas*) within *Proteobacteria* and *Firmicutes* might be capable of post-depositional biological production of N_2_O *in situ* at some depths of the North Greenland Eemian Ice Drilling ice core (Miteva et al., 2016). These reports suggested that excess gases (i.e., CO_2_, CH_4_, and N_2_O) at some depths in the ice cores are due to ongoing *in situ* production by microorganisms. However, microbial activity is presumed to be very low in the deep glacial ice (Maccario et al., 2015). Furthermore, there is no direct evidence to indicate that microorganisms are active *in situ* in the ancient ice cores.

These advances have come in spite of glacier ice being a challenging medium in which to study microbial communities. First, microbial biomass in glacier ice is low (cell concentrations range from 10^2^ to 10^4^ cells ml^-1^) and often only small volumes of ice are available (Miteva, 2008). Second, it is difficult to disrupt spore-forming and non-sporulating Gram-positive cells, which are frequently detected in glacier ice cores (Christner et al., 2000; Abyzov et al., 2004; Steven et al., 2008; Knowlton et al., 2013). These problems hamper obtaining microbial DNA of sufficient quantity and quality for culture-independent studies. In addition, because of its low biomass, contamination from sampling, storage, and preparation conditions is a major issue for studies of microbial communities in ice (Ram, 2009).

The surface ice of the ice core probably contains microbial contaminants that were introduced during drilling or handling ice cores in the fields or labs. Therefore, it is important to remove microbial contaminants on the surface of glacier ice cores (surface decontamination) for collecting low-contaminant ice samples. Considerable effort has been put forth to develop clean sampling technology and a number of surface decontamination strategies have been proposed and summarized in detail (Rogers et al., 2004; Christner et al., 2005). Briefly, these methods either killed microorganisms with chemical regents (Rogers et al., 2004), washed and removed the microorganisms in surface ice (e.g., Karl et al., 1999; Priscu et al., 1999; Christner et al., 2005), or collected the ice core interior by using a melting device (e.g., Abyzov, 1993; Christner et al., 2000). After surface decontamination, microbial contaminants can also be introduced into ice samples from environments (e.g., laboratory personnel, tools, reagents, and air) during the processing of ice including ice sampling, concentrating cells, and DNA extraction and sequencing. Specifically, DNA extraction methods can have a profound effect in studying microbial communities, and it has been a major source of variation in microbial Metagenomic work for low-biomass samples (Morgan et al., 2010; Woyke et al., 2011; Salter et al., 2014; Glassing et al., 2016). “Background” controls help reveal potential contaminants introduced during the processing of ice after surface decontamination (Willerslev et al., 2004; Hebsgaard et al., 2005), and the studied sample datasets can be *in silico* decontaminated by removing microbiota found in “background” controls. “Background” controls were included for microbial investigations of glacier ice in some reports, whereas some found that these controls did not yield any amplification products and suggested “clean” ice processing procedures (Sheridan et al., 2003; Yao et al., 2008; Zhang et al., 2008; Liu et al., 2009). Open air culture plates were used to check potential air contaminants that could be cultivated and were removed from the ice samples (Ram, 2009; Knowlton et al., 2013; Miteva et al., 2015). Two “background” controls (nanopure water and autoclaved nanopure water) were conducted during a metagenomic study of two ice samples from the Greenland Ice Sheet Project 2 ice core (Knowlton et al., 2013). A total of 55,254 and 52,078 high-quality 454 reads were generated for two ice and two control samples, respectively. Only 33 sequences that were unique to the ice were selected for further microbial analysis after removing the sequences that were in common with the control samples and were considered as potential contaminants (Knowlton et al., 2013). In another study, nine ice samples were excluded from further microbial analysis since they had a high abundance (68.7 ± 24.8%) of an operational taxonomic unit (OTU) that was also abundant (73.1%) in a “background” control sample conducted in parallel to the ice DNA extractions (Cameron et al., 2016).

All of these studies removed suspected “contaminants” in ice samples by conducting “background” controls and obtained decontaminated data for further microbial analysis. It is a challenge to determine if the removed microorganisms were from ice or contaminants, and it has been suggested to not remove OTUs identified in “background” controls due to cross-contamination if they are biologically expected in the given sample type (Salter et al., 2014).

Here we sought to establish low-biomass, culture-independent “clean” procedures to survey microorganisms in glacier ice and then together with several “background” controls and published *in silico* decontamination methods use them to identify and quantify microbial diversity at two depths in an ice core from the Guliya ice cap in northwestern Tibet. The Tibetan Plateau is a mountainous area (average altitude of ∼4,500 m) that covers about 2.5 million km^2^ of the Eurasian continent (Cui and Graf, 2009). It contains the third largest reservoir of glacial ice on Earth (Qiu, 2008) and is the major water source for Southern and Eastern Asia (Cui and Graf, 2009; Immerzeel et al., 2010). The Guliya ice cap is located at the northwestern Kunlun Mountains of the Tibetan Plateau and is the highest (6,700 m), largest (>200 km^2^), thickest (308.6 m), and coldest (-18.6°C) ice cap among all the ice caps in middle-low latitude regions (Yao et al., 1992; Thompson et al., 1995). Previous studies on the Guliya ice cap focused primarily on the formation, structure, geochemistry, and dating of the ice, and found that the Guliya ice cap preserved the history of past climate change over tens to hundreds of thousands of years (Yao et al., 1992, 2004; Thompson et al., 1995, 1997, 2000; Wang et al., 2002; Wu et al., 2004). The microbial community in this ice cap has been largely unexplored except for two culture-dependent studies, which recovered viable bacterial strains immured in glacial ice that was more than 500,000 years old. These recovered isolates belonged to the alphaproteobacterial, betaproteobacterial, actinobacterial, and low-G+C Gram-positive bacterial lineages (Christner et al., 2000, 2003).

## Materials and Methods

### Site Characterization and Field Sampling

**FIGURE 1:**
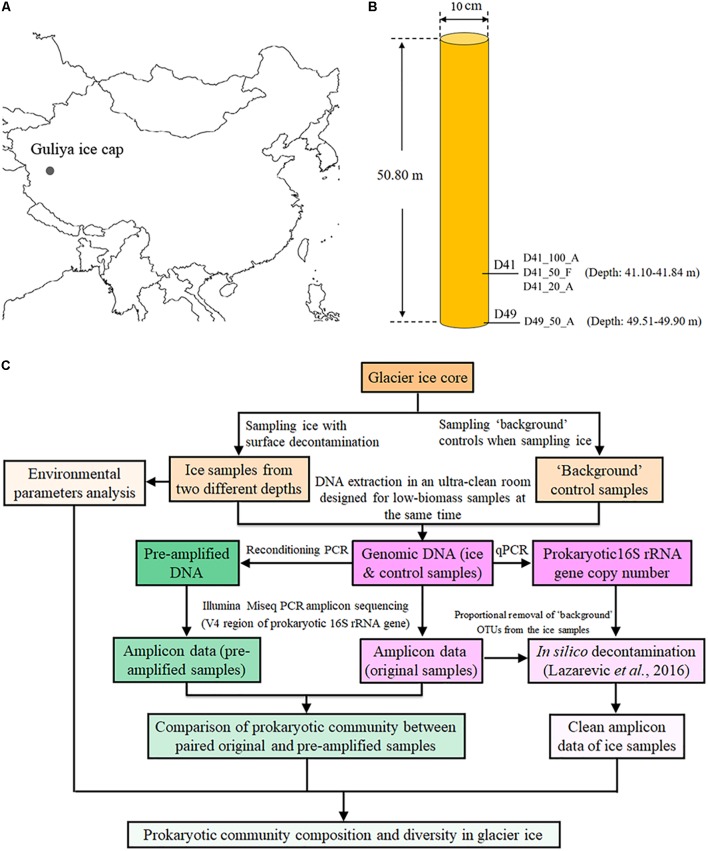
Location **(A)**, sampling sites **(B)**, and an overview of experimental design **(C)** for investigating the microbial communities of the GS3 ice core drilled from the Guliya ice cap. The sample names from this study are coded as follows for the example of D41_100_A: D41, the depth of the ice sample (41 m under the surface); 100, the ice volume for DNA extraction (100 ml). Abbreviations of two methods for concentrating cells: A, Amicon Ultra Concentrators; F, filters with 0.22-μm pore size. Three samples including D41_100_A, D41_50_F, and D41_20_A were collected from the same mixture of melted ice from 41.40 to 41.84 m deep, while D49_50_F was sampled from ice 49.51–49.90 m deep.

The Guliya summit 3 (GS3) ice core was drilled in October 2015 from the summit of the Guliya ice cap (35°17′ N, 81°29′ E, ∼6700 m above sea level, **Figure 1A**). This ice core was 10 cm in diameter, 50.80 m in length (**Figure 1B**), and the bedrock temperature was about -15°C. Ice core sections were sealed in plastic tubes, put into cardboard tubes covered with aluminum, and transferred at -20°C by truck from the drill site to freezers in Lhasa, by airplane to freezers in Beijing, by airplane to Chicago, and then by freezer truck to the Byrd Polar and Climate Research Center at Ohio State University where it is stored at -34°C.

### Ice Core Sampling and Physiochemical Conditions

The GS3 ice core sections were transferred from -34°C to the sampling temperature of -5°C overnight to reduce the possibility of fracturing during surface decontamination by cutting and washing. The decontamination procedures used included washing and removing the surface of the ice core as described previously (Karl et al., 1999; Priscu et al., 1999) with some modifications that added an additional removal of the ice core’s outermost layer by cutting with a band saw. Briefly, ∼6 mm of the outermost layer was removed from the ice cores with a band saw. The inner ice core was cut into 3–4 cm sections in a cold room (-5°C) and the sections were thoroughly washed with filtered (0.22-μm-pore-sized filter) and sterilized water to remove 3–5 mm of the surface layer after which they were melted in covered containers in a Class 100 clean room at room temperature for about 4 h. Although prior bacterial cultivation work was conducted in the same cold and clean rooms, no biological experiments of any kind had been conducted in them for more than 10 years. Sections of melted ice from the depth of 41.10–41.84 m of the GS3 ice core were combined as one sample (D41), and those from 49.51 to 49.90 m were combined as another sample (D49; **Figure 1B**), for microbial analysis. Dust, chemical ions, and oxygen isotopes were analyzed as described previously (Davis and Thompson, 2006). The approximate age of each ice section was dated by matching the oxygen isotopic ratios with those from another 310.6-m ice core of similar age collected from the Guliya ice cap in 1992 (Thompson et al., 1995, 1997).

### “Background” Controls

Four “background” control samples were used to investigate possible sources of background contamination during processing. First, we assessed what microorganisms were in the air from the cold and clean rooms used for ice core processing. Specifically, cells from 28.3 and 28.8 m^3^ of air were collected from the cold room (named Air_ColdRoom) and the clean room (Air_CleanRoom), respectively. Cell collection in the air started at the same time as the processing of the GS3 ice core sections, and continued after ice core processing for a total of 4 days of sampling. The air samples were passed through sterilized polycarbonate 0.8-μm-pore-sized filters (Cat No. ATTP02500, Isopore), as well as a Button Aerosol Sampler (SKC Inc.), which is reported to have higher recovery efficiency of bacteria (specific recovery efficiency not provided) in indoor and outdoor air compared to three other samplers including the IOM Inhalable Dust Sampler, the NIOSH Personal Bioaerosol Cyclone Sampler, and the 37-mm Filter Cassette sampler (Wang et al., 2015). These two controls evaluated background contamination due to exposure to air during the ice processing. Second, an artificial ice core made from 0.22-μm filtered (Cat No. MPGP04001, Millipak^®^ Express 40 Filter, Merck KGaA) and autoclaved (121°C for 30 min) water was frozen (-34°C for 12–24 h) and then processed in parallel with the GS3 ice core samples through the entire analysis. This control facilitated evaluation of contamination from the instruments used to process the ice. Finally, a blank control was established by extracting DNA directly from 400 ml of 0.22-μm filtered and autoclaved water (as above). This control allowed evaluation of contamination downstream of the ice processing, including the molecular procedures (DNA extraction, PCR, library preparation, and sequencing).

### Genomic DNA Extraction

A total of 400 ml of artificial ice (Artificial_ice), 400 ml of the blank control (Blank), and 50 ml each of the two ice samples (D41_50_F and D49_50_F) were filtered through sterilized polycarbonate 0.22-μm-pore-sized filters (Cat No. GTTP02500, Isopore) to collect microorganisms including all bacterial/archaeal cells, with cell sizes exceeding 0.22 μm. The filters were used to isolate DNA. DNA was also isolated from cells concentrated from 100 (D41_100_A) and 20 ml (D41_20_A) of Sample D41 to 0.6 ml by 100 kDa Amicon Ultra Concentrators (EMD Millipore, Darmstadt, Germany), with a pre-filtration by 3.0-μm-pore-size filters to remove big dust particles to avoid clogging the concentrators. Community DNA was isolated from these four ice samples (D41_100_A, D41_20_A, D41_50_F, and D49_50_F) and the four “background” controls (Artifical_ice, Blank, Air_ColdRoom, and Air_CleanRoom) with a DNeasy Blood & Tissue Kit (Cat No. 69506, QIAGEN) according to the manufacturer’s instructions, with an additional step of beating with beads to disrupt bacterial spores and Gram-positive cells before cell lysis by homogenizing at 3,400 rpm for 1 min with 100 mg of autoclaved (121°C for 30 min) 0.1-mm-diameter glass beads (Cat No. 13118-400, QIAGEN) in a MiniBeadBeater-16 (Model 607, BioSpec Products). DNA was stored at -80°C. DNA denaturants (DNA AWAY, Cat No. 7010, Thermo Scientific) and 70% ethanol were used to eliminate potential naked DNA and cell contaminants on the surface of gloves, lab benches, and some tools used in this study.

### Real-Time Quantitative Polymerase Chain Reaction (qPCR)

Total bacterial and archaeal biomass was estimated using real-time qPCR for the four ice samples and the four “background” controls after isolating DNA. Primer sets 1406f (5′-GYACWCACCGCCCGT-3′) and 1525r (5′-AAGGAGGTGWTCCARCC-3′) were used to amplify bacterial and archaeal 16S rRNA genes (Vanwonterghem et al., 2014). Each 20-μl reaction contained: 10 μl 2× QuantiTect SYBR Green PCR Master Mix (Cat No. 204143, QIAGEN), 0.5 μl of each primer (1406f/1525r, 10 mM), 3 μl template DNA, and 6 μl RNase-free water. Thermocycling consisted of an initial polymerase activation and template DNA denaturation at 95°C for 15 min, followed by 40 cycles of 95°C for 15 s, 55°C for 30 s, and 72°C for 15 s. A melt curve was produced by running a cycle of 95°C for 15 s, 55°C for 15 s, and 95°C for 15 s. A standard curve was generated with a PCR product using primers 1406f/1525r from *Cellulophaga baltica* strain 18 (NCBI accession number of the complete genome, CP009976). All reactions were performed in triplicate, using an Illumina Eco cycler (Cat No. 1010180).

### Reconditioning PCR

Reconditioning PCR, reported to reduce PCR artificial bias (Thompson et al., 2002; Lenz and Becker, 2008), was conducted for each sample to pre-amplify the V4 region of prokaryotic 16S rRNA genes with primer sets 515f/806r (Caporaso et al., 2011), which was selected for amplicon sequencing to investigate the microbial community. A Phusion High-Fidelity DNA Polymerase Kit (Cat No. F530L, Thermo Scientific) was used for reconditioning PCR. The 20 μl PCR reaction consisted of: 4 μl 5× Phusion HF Buffer (containing MgCl_2_), 0.4 μl 10 mM dNTP, 1 μl of each primer (515f/806r, 10 mM), 0.2 μl high-fidelity DNA polymerase, 2 μl template DNA, and 11.4 μl of water. For all eight samples, the first round amplification consisted of a 40-s denaturing step at 98°C, followed by 28 cycles of 8 s at 98°C, 20 s at 48°C, and 15 s at 72°C, with a final extension of 8 min at 72°C. To recondition the PCR products, the amplified reactions were diluted fivefold into a fresh reaction mixture of the same composition and cycled eight times using the same conditions as the first round PCR. All reactions were conducted in triplicate, which were combined as one sample after each PCR. The combined reaction mixtures after reconditioning PCR were purified by Agencourt AMPure XP Beads (Cat No. A63881, Beckman Coulter) and collected in 50 μl of buffer, according to the manufacturer’s instructions.

### Tag-Encoded Amplicon Pyrosequencing of Microbial Community

Bar-coded primers 515f/806r (Caporaso et al., 2011) were selected to amplify the V4 hypervariable regions of 16S rRNA genes of bacteria and archaea for both original and pre-amplified samples. Resulting amplicons were sequenced by the Illumina MiSeq platform (paired-end reads), as described previously (Caporaso et al., 2011, 2012). These experiments were performed at Argonne National Laboratory.

### Sequence Analysis

Sequences with an expected error >1.0 or length <245 nt were excluded from the analyses (Edgar, 2013). The remaining sequences were truncated to a constant length (245 nt). Various analyses were conducted using the Quantitative Insights Into Microbial Ecology (QIIME, version 1.9.1) software package (Caporaso et al., 2010) with default parameters, except that chimera filtering, OTU clustering, and singleton excluding were performed with QIIME through the UPARSE pipeline (Edgar, 2013). A phylogenetic tree was constructed with a set of sequence representatives of the OTUs using the method of FastTree (Price et al., 2009). Chimeras were identified and filtered by UPARSE with the UCHIME algorithm using the ChimeraSlayer reference database (Haas et al., 2011), which is considered to be sensitive and quick (Edgar et al., 2011). Reads were clustered into OTUs at 97% sequence similarity by UPARSE. A representative sequence from each OTU was selected for taxonomic annotation using the Ribosomal Database Project (RDP) classifier (Wang et al., 2007) from the RDP Release 11.5 database. Taxonomic assignments with <80% confidence were marked as unclassified taxa. Mitochondrial and chloroplastic sequences were excluded from further analysis.

Relative abundance of the microbial profiles at the genus level was calculated for each sample. The differences in microbial community composition between each paired original and pre-amplified samples were tested for significance using a two-tailed paired *t*-test. A heatmap was generated based on the number of sequences per OTU per 30,000 sequences using functions in the Pheatmap package version 1.0.8 (Kolde, 2015) in R version 3.4.2 (R Core Team, 2012). A new profile of OTU composition for the ice samples was generated after *in silico* decontamination as described previously (Lazarevic et al., 2016). Briefly, an R-OTU value was designated as the ratio between the mean “absolute” abundance of OTUs in “background” controls and ice samples; then, an approximated estimation of the “absolute” abundance of OTUs was calculated by multiplying the relative abundance of each OTU by the 16S rRNA gene copy number in a given sample (determined by qPCR). The OTUs with R-OTU values >0.01 were considered to be contaminants and were removed from the ice samples. The significance of the difference in microbial community between D41 and D49 samples was evaluated by Analysis of Similarity Statistics (ANOSIM) (Clarke, 1993), which was performed using functions in the Vegan package version 2.4-4 (Dixon, 2003) in R version 3.4.2 (R Core Team, 2012).

### Comparison of Microbial Profiles Between Guliya Ice Cap and Several Other Ice Caps

The microbial profiles of the Guliya ice cap were compared to those from other glaciers and ice fields previously characterized by next-generation sequencing of the overlapped region (V4) of the 16S rRNA gene. The selected samples included two to four samples from each of three Tibetan Plateau ice caps (Geladangdong, Noijinkangsang, and Zuoqiupu) (Liu et al., 2016) and the Greenland ice sheet (Miteva et al., 2015, 2016). Sequence files (.fastq) of each sample were obtained from NCBI Sequence Read Archive using the SRA Toolkit^1^ and combined with that of the Guliya ice cap samples from this study. Sequences were analyzed as described in the previous section. In addition, samples were also clustered by the unweighted pair group method with the arithmetic mean (UPGMA) based on weighted UniFrac distances, which accounts for changes in relative taxon abundance (Caporaso et al., 2010). Principal coordinates analysis (PCoA) using weighted UniFrac metrics was performed to distinguish general distribution patterns of microbial profiles among all samples. The Mantel test was conducted to evaluate the linkage between the microbial community structure and environmental parameters.

### Nucleotide Sequence Accession Numbers

The nucleotide sequences discovered during this study have been deposited in the NCBI Sequence Read Archive under accession number SRP114723.

## Results and Discussion

A GS3 ice core, which was 50.80 m in length and contained ice up to ∼30,000 years old, was retrieved from the Guliya ice cap, China in 2015 (**Figures 1A,B**) to monitor past climate change and archived microbial profiles. In this study, four “background” controls including a sterile water artificial ice core (named as Artificial_ice), two air samples collected from the ice processing laboratories (Air_ColdRoom and Air_CleanRoom), and a blank sterile water sample (Blank) were co-processed with four real ice samples to check “background” microbial profiles and their abundances, and establish “clean” sampling and amplicon sequencing protocols. Subsequently the procedures, together with published *in silico* decontamination methods, were used to investigate the microbial profiles archived in ice at two depths in the GS3 ice core (overview in **Figure 1C**).

### Establishment of Microbial “Contaminants” From Four “Background” Controls

To obtain clean amplicon sequencing reads of ice samples, the first step was to identify how much biomass and what microbial taxa (contaminants) were in the four “background” controls. Total microbial cell abundance was first measured by epifluorescence microscopy after the cells were concentrated on a 0.22-μm-pore-sized filter (Cat No. GTTP02500, Isopore) and stained by SYBR Gold as described previously (Noble and Fuhrman, 1998). A total of 11.3 × 10^3^ and 8.4 × 10^3^ cells were observed for Air_ColdRoom and Air_CleanRoom, respectively; while less than 100 and 10 cells were detected on the filters of Artificial_ice and Blank, respectively (data not shown). The DNA extraction process could also introduce contaminations, such as those from investigators (e.g., human skin and respiratory), tools, and reagents (Woyke et al., 2011; Salter et al., 2014). Therefore, qPCR was performed to assess how much biomass of microbial DNA was obtained from the four “background” controls after DNA extraction, by calculating the copy number of 16S rRNA genes with reference to a standard curve. The 16S rRNA gene copies per microliter in the 50-μl volumes of each sample revealed 49, 49, 51, and 27 copies/μl in Air_ColdRoom, Air_CleanRoom, Artificial_ice, and Blank, respectively (Supplementary Figure S1).

**FIGURE 2:**
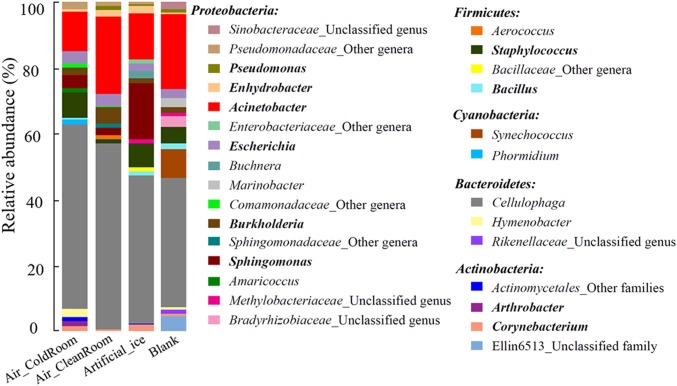
Microbial community structure of the 29 most abundant genera in the four “background” controls. Genera belonging to the same phylum are described under the phylum name. The “other genera/families” represent unclassified sequences and could not be assigned to a single genus/family. Genera previously reported as contaminant taxa are indicated in bold. The four “background” controls: Air_ColdRoom and Air_CleanRoom, two air samples collected from a cold and clean room, respectively, in which the ice samples were processed; Artificial_ice, an artificial ice sample made by sterile water and processed along with the glacier ice samples; Blank, a blank sample with 400 ml sterile water.

Microbial profiles in the four “background” controls were investigated using Illumina Miseq PCR amplicon sequencing. The QC data were normalized to 15,000 sequences for each sample (i.e., each MiSeq sequencing library) for further analysis. These sequences were affiliated with 169 bacterial genera, 94 of which had recognized names (Supplementary Table S1). The 29 most abundant genera, each of which accounted for ≥1.0% of the sequences in at least one sample, comprised 82.9–88.8% of each community and were selected to illustrate the microbial communities of the four “background” controls (**Figure 2**). These genera belonged to five phyla, *Proteobacteria*, *Firmicutes*, *Cyanobacteria*, *Bacteroidetes*, and *Actinobacteria*, which contained 16, 4, 2, 3, and 4 genera, respectively (**Figure 2**). Many exogenous sequences assigned as unexpected taxa from contamination have been detected during the analysis of low-biomass environmental microbiota (Biesbroek et al., 2012; Lazarevic et al., 2014), cultures (Salter et al., 2014; Lazarevic et al., 2016), and diluted mock microbial communities (Willner et al., 2012). These contaminants might come from lab air (Othman, 2015; Lauder et al., 2016), investigators (e.g., human skin and respiratory) (Knights et al., 2011), tools, and reagents used for DNA extraction, PCR amplification, and sequencing (Corless et al., 2000; Barton et al., 2006; Glassing et al., 2016). Some contaminant genera detected in this study overlapped with previously described contaminant groups, including the genera *Sphingomonas* (Barton et al., 2006; Laurence et al., 2014), *Burkholderia* (Laurence et al., 2014), *Escherichia* (Tanner et al., 1998; Laurence et al., 2014; Salter et al., 2014), *Acinetobacter* (Tanner et al., 1998; Barton et al., 2006), *Enhydrobacter* (Salter et al., 2014), *Pseudomonas* (Grahn et al., 2003), *Corynebacterium* (Salter et al., 2014), *Arthrobacter* (Salter et al., 2014), *Bacillus* (Grahn et al., 2003), and *Staphylococcus* (Othman, 2015; **Figure 2**). These findings indicate that many microbial taxa are common contaminants in microbial community studies. Two additional genera, *Cellulophaga* and *Synechococcus*, were detected and interpreted as contaminants in this study (**Figure 2**). Many isolates belonging to these two genera have been used previously as type strains to investigate virus–host interactions in our laboratory (Deng et al., 2014; Dang et al., 2015), which is why we interpreted these as low-level laboratory contaminants. It is likely then that such contamination would vary from laboratory to laboratory for low biomass samples, which is consistent with prior findings (Willerslev et al., 2004).

### Paired Original and Pre-amplified Samples Capture Almost Identical Microbial Profiles

A previous report (Salter et al., 2014) indicated that it is difficult to determine the composition of a microbial community if the number of microorganisms used for DNA extraction is less than 10^3^–10^4^ cells. Considering the low biomass in our “background” controls (10^0^–10^4^ cells), we pre-amplified the targeted region (V4) of bacterial and archaeal 16S rRNA genes by reconditioning PCR (Thompson et al., 2002) in all of the four original “background” controls before standard amplicon sequencing. These pre-amplified samples were subjected to standard amplicon sequencing together with the original “background” controls. Microbial profiles were compared between each pair of original and pre-amplified samples to determine whether reliable microbial community values were obtained for the original and their pre-amplified “background” controls.

All of the four original and four pre-amplified libraries were normalized to 15,000 sequences for further analysis. The 36 most abundant genera, each of which accounted for >1.0% of sequences in at least one sample, comprised 85.7% of the total 120,000 sequences in eight samples. These groups were designated as “major genera” and used to exemplify the microbial community of all the original and pre-amplified samples (Supplementary Table S2 and Supplementary Figure S2). All of these “major genera” were detected in each pair of original and pre-amplified samples, and accounted for almost all of each microbial community (Supplementary Table S2 and Supplementary Figure S2). For example, the 16 most abundant genera, including *Cellulophaga*, *Acinetobacter*, *Staphylococcus*, *Sphingomonas*, *Escherichia*, *Hymenobacter*, *Burkholderia*, an unclassified genus within the family *Pseudomonadaceae*, *Corynebacterium*, *Arthrobacter*, *Amaricoccus*, an unclassified genus within the family *Comamonadaceae*, *Enhydrobacter*, *Propionibacterium*, *Stenotrophomonas*, and *Streptococcus*, were all similarly represented in the original sample Air_ColdRoom and its pre-amplified sample Air_ColdRoom_28+8 (“28+8” represents 28 and 8 cycle times at the first and the reconditioning PCR rounds, respectively) (Supplementary Table S2 and Supplementary Figure S2). These 16 genera comprised 96.7 and 95.6% of the microbial community in Air_ColdRoom and Air_ColdRoom_28+8, respectively. In addition, results from the two-tailed paired *t*-test showed pre-amplification with reconditioning PCR does not significantly alter the microbial community in original samples (*p*-values were 0.60–0.92 for the above four pairs of original and pre-amplified samples, respectively). The similar community composition in each pair of original and pre-amplified samples indicates that the reliable microbial profile values were obtained for both original and pre-amplified “background” controls, and that reconditioning PCR captures a microbial community that is almost identical to the original samples with low biomass. Lenz and Becker (2008) used standard PCR and reconditioning PCR to analyze polymorphic loci and investigate genetic variation in the major histocompatibility complex (MHC) class IIB genes of the three-spined stickleback (*Gasterosteus aculeatus*). They reported that 24% of the clones were artificial allele chimeras generated by the hybrids of two or three different alleles that occurred in the same individual, using standard PCR, while the number of artificial chimeras was reduced 10-fold by reconditioning PCR (Lenz and Becker, 2008). The results from this study and previous reports confirm that reconditioning PCR reduces amplification bias from multi-template PCR products before library construction and the results more closely reflect the genetic diversity of the original samples (Thompson et al., 2002). In addition, our previous studies of viromes suggest that the degree of amplification has little impact on the resulting metagenomes (Duhaime et al., 2012; Solonenko et al., 2013; Solonenko and Sullivan, 2013).

### Proportional Removal (*In Silico* Decontamination) of “Contaminants” From the GS3 Ice Core Samples

With the established contaminant taxa from the four “background” controls, we next *in silico* removed these contaminants in the amplicon sequencing dataset of the four Guliya ice samples to generate “clean” sequencing reads of these ice samples by the following procedure. A recently published *in silico* decontamination strategy, that combines the information of both the relative abundance of each OTU and the 16S rRNA gene copy number in a given sample (proportional removal), effectively removes the contaminant sequences derived from the “background” controls in the samples of interest (i.e., ice samples) as described in the section “Materials and Methods” (Lazarevic et al., 2016). To use this *in silico* decontamination strategy for the ice samples and the “background” controls, we first quantified the 16S rRNA gene copy number in the ice samples and checked the differences in the OTU compositions between the ice samples and the “background” controls.

**FIGURE 3:**
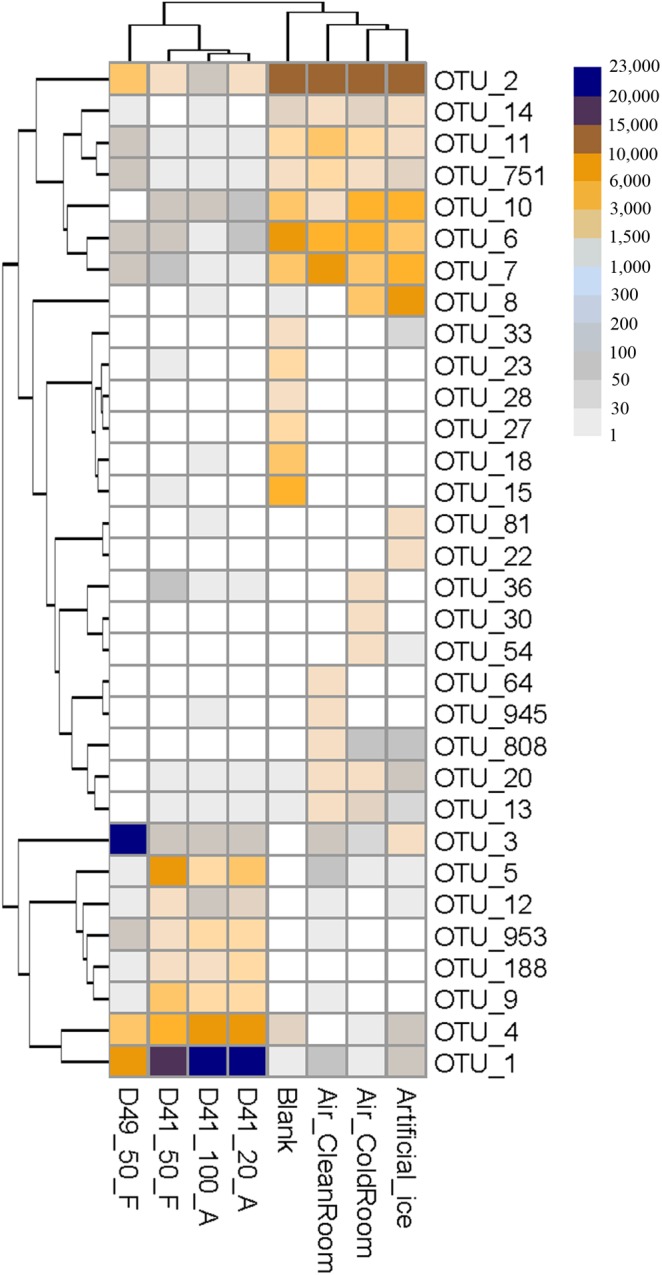
Heatmap showing the sequence number of each OTU per 30,000 sequences for the Guliya ice samples and “background” controls. All OTUs accounted for >1.0% of sequences (i.e., >300 sequences) in at least one sample. OTUs were defined as reads with 97% sequence similarity.

The 16S rRNA gene copies per microliter in the 50 μl volumes from each ice sample were 4.60 × 10^3^, 0.97 × 10^3^, 0.95 × 10^3^, and 1.25 × 10^3^ copies/μl in D41_100_A, D41_20_A, D41_50_F, and D49_50_F, respectively (Supplementary Figure S1). Thus the biomass in the ice samples was about 50–100-times higher than that in all four “background” controls (27–51 copies/μl, Supplementary Figure S1). The amplicon data of the four Guliya ice samples and four “background” controls were normalized to 30,000 sequences for further analysis. The 32 most abundant OTUs (relative abundance was >1.0% in at least one sample) comprised 88.6% of the total sequences (240,000) of the ice samples and the “background” controls, and were selected to illustrate their OTU compositions (**Figure 3**). Total sequences belonging to eight OTUs, including OTU_1, OTU_4, OTU_5, OTU_9, OTU_3, OTU_953, OTU_188, and OTU_12, accounted for 93.4–98.9% of all sequences in the 32 OTUs for each ice sample, but only made up 0.3–2.9% of the “background” controls (**Figure 3**). In contrast, the other 24 OTUs contributed 1.1–6.4 and 97.1–99.7% of the sequences in the 32 OTUs from the ice samples and “background” controls, respectively (**Figure 3**). These results indicate that the most abundant OTUs in the ice samples were notably different from those in the “background” controls, and that the latter 24 OTUs are probably contaminants and should be *in silico* removed from the ice samples before taxonomic analysis. Sequences belonging to the most abundant OTUs in the ice samples (i.e., OTU_1, OTU_3, and OTU_4) were also detected in small amounts in the “background” controls (**Figure 3**). Similar results were also observed in a study that investigated the bacterial community in mock and control samples (Lazarevic et al., 2016), indicating possible cross-contamination during DNA extraction from samples with much higher biomass to samples with lower biomass (e.g., from ice to “background” controls in this study). Thus, special caution should be taken with regard to the suspicious “contaminating” microorganisms that are also discovered to be present in the investigated environments.

The dataset of ice samples in this study was decontaminated *in silico* with the proportional removal strategy mentioned above using R-OTU cut-off values of 0.01 by removing OTUs with this ratio exceeding 0.01 (Lazarevic et al., 2016). After *in silico* decontamination, 93.2–97.8% of the reads in the ice samples were retained, while only 0.2–2.3% of the reads were retained in the “background” controls; this small number of reads might represent cross-contamination of the ice samples with much higher biomass (Supplementary Figure S1), as discussed above. An important but largely unrecognized source of laboratory-based contamination is PCR product carryover because the amount of contaminant DNA might be larger than the DNA in the glacier ice samples (Kwok and Higuchi, 1989; Willerslev et al., 2004; Willerslev and Cooper, 2005). DNA molecules and cells from laboratory personnel, tools, reagents, and air can also introduce contaminants. Thus, it is important to include no-template control “blank” samples in experiments with low biomass to control for this low-level source of contamination (Hebsgaard et al., 2005). “Background” controls and the subsequent removal of “suspect” contaminants were included in some prior culture-dependent and -independent microbial studies with glacier ice cores (Ram, 2009; Knowlton et al., 2013; Miteva et al., 2015; Cameron et al., 2016). These reports reflect the laboratory contamination in the glacier ice samples and indicate the necessity to *in silico* remove the contaminants. The challenge is to determine if the removed microorganisms originated from ice or contaminants. The proportional removal approach used in this study may efficiently find the OTUs derived from cross-contamination, in contrast to those derived from reagents, and thus not remove them from the dataset; this process may improve the taxonomic representation in the low-biomass ice samples.

We note, however, that there are variations across taxa in DNA extraction and recovery efficiency (Yuan et al., 2012), which is associated with the qPCR-quantified 16S rRNA gene abundance in this study. Our method can be used to proportionally adjust the contaminants based on their relative amounts if the DNA extraction and recovery efficiency are similar or nearly identical for the same taxon across samples. We also realize that it is hard to quantify the amount of contamination from air to ice samples, and that the volume of collected air or other factors also influence the 16S rRNA gene concentration that is used to calculate the “absolute” abundance of each OTU. The retrieved biomass of air samples was only a small fraction of that observed in the ice samples (Supplementary Figure S1), although the air was continuously sampled for 4 days. This suggests that the cold and clean rooms were quite clean for processing the low-biomass ice samples in this study. The DNA-denaturing regent was used to “clean” the surface of the bench, gloves, and some tools before processing the glacier ice samples, but not used for removing naked DNA from the filtered and/or autoclaved water, or from reagents we used. However, the “background” controls can help identify and remove such contamination *in silico* from any possible contaminant naked DNA in the ice. With more attention paid to conducting “background” controls and *in silico* decontamination for microbial investigations of low-biomass glacier ice, as well as the usage of internal standards to better control for DNA extraction and recovery efficiency (Mumy and Findlay, 2004), we will be able to remove “background” contaminants more efficiently and obtain cleaner ice microbial data in future studies.

### Microbial Profiles Differ Between Ice Samples From Two Different Depths of the GS3 Ice Core

**FIGURE 4:**
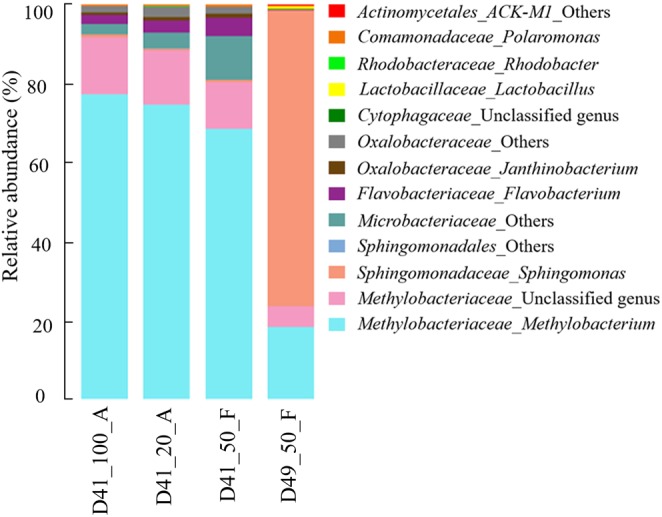
Microbial community structure of the 13 most abundant genera in the GS3 ice core samples. The “others” represent unclassified sequences and could not be assigned to a single genus.

With the “clean” reads after *in silico* decontamination, we then examined the microbial communities of three and one ice samples collected from 41 and 49 m depths in the GS3 ice core, respectively. These “clean” reads in the four ice samples contained 169 bacterial genera, 70 of which had recognized names (Supplementary Table S3). The 13 most abundant genera, each of which accounted for >0.1% of sequences in at least one ice sample, comprised >98.5% of each decontaminated community. These genera were selected to illustrate the microbial community structures of the four ice samples (**Figure 4**). We also pre-amplified the targeted region (V4) of prokaryotic 16S rRNA genes by reconditioning PCR (Thompson et al., 2002) in all four of the ice samples before standard amplicon sequencing, as conducted for the “background” controls. The community compositions in each pair of original and pre-amplified ice samples were indistinguishable (Supplementary Figure S3). These results indicate that reliable microbial profile values were captured for both original and pre-amplified ice samples. The relative abundances of the microbial community among the three D41 samples (i.e., D41_100_A, D41_20_A, and D41_50_F) showed no significant difference from one another based on the results from the two-tailed paired *t*-test (*p*-values were 0.85–0.99, **Figure 4**). In addition, the results from ANOSIM analysis (together with their pre-amplified samples D41_100_A_28+8, D41_20_A_28+8, and D49_50_F_28+8 shown in Supplementary Figure S3) confirmed that the microbial communities in group samples (e.g., D41_100_A and D41_100_A_28+8 were a group) were not significantly different from one another (*p*-values were 0.66–0.99, *n* = 999). These results indicate that similar microbial profiles were captured from the ice samples regardless of the differences in sample volume and concentrating methods used for collecting cells and DNA extraction.

The genus *Methylobacterium* within the family *Methylobacteriaceae* was the most abundant taxon in the three D41 samples and had a relative abundance of 67.3–76.6%. An unclassified genus belonging to the same family, *Methylobacteriaceae*, was also abundant (relative abundance 11.7–14.3%) in these three samples (**Figure 4**). Members belonging to the genus *Methylobacterium* were also reported to dominate the microbial community in ancient ice cores from many previous studies (Miteva, 2008; Segawa et al., 2010; Antony et al., 2012; Miteva et al., 2015, 2016), including several microbial investigations of the Guliya ice cap ice cores using culture-dependent methods (Christner et al., 2000, 2001; Christner, 2003). Five other genera with relative abundances of 0.1–4.5%, which had recognized names, were also previously reported to be abundant in glacier ice cores, including the genera *Flavobacterium* (Liu et al., 2015; Chen et al., 2016), *Janthinobacterium* (Christner, 2003; Miteva, 2008), *Polaromonas* (Liu et al., 2009; An et al., 2010; Chen et al., 2016), *Sphingomonas* (An et al., 2010; Miteva et al., 2016), and *Rhodobacter* (Liu et al., 2015). The detection of bacterial sequences belonging to similar genera in ice core samples from different glaciers located around the world can be explained by the ubiquitous distribution of certain species in geographically distant environments (Baas Becking, 1934; Martiny et al., 2006). Furthermore, many *Methylobacterium* and *Sphingomonas* members are commonly found in tropospheric clouds and concentrated in cloud water (Amato et al., 2007; DeLeon-Rodriguez et al., 2013), which would allow them to be deposited onto the glaciers with falling snow.

For sample D49_50_F, the genus *Methylobacterium* and the unclassified genus (same as in D41 samples) within the family *Methylobacteriaceae* were also abundant making up 18.3 and 5.2%, respectively, of the total microbial population (**Figure 4**). The most abundant genus in this sample, however, was *Sphingomonas* with a relative abundance of 75.2%. Three other genera, including *Lactobacillus* and two unclassified genera in the phyla *Bacteroidetes* and *Actinobacteria*, accounted for 0.1–0.7% of the sequences (**Figure 4**). Thus there is a notable difference in the microbial profiles between samples D41 and D49. The results from ANOSIM analysis confirmed that the microbial communities were significantly different between samples from D41 and D49 (*p* = 0.04, *n* = 999).

Previous studies have often reported different microbial community structures in ice samples collected from different depths of the same ice core, and this probably reflects differences in the environmental conditions among ice samples (Priscu et al., 2007; Miteva et al., 2015; Liu et al., 2016). The D41 and D49 samples were obtained from depths of 41.10–41.84 and 49.51–49.90 m of the GS3 ice core, respectively (**Figure 1B** and Supplementary Table S4). These samples are approximately 20,000 and 30,000 years old, respectively (Supplementary Table S4), as determined by preliminary matching of the GS3 stable oxygen isotopes with those in a 1992 Guliya ice cap ice core (Thompson et al., 1997). The concentrations of nitrogen-related ions NO_3_^-^ and NH_4_^+^ in D49 were lower than those in D41 while higher concentrations of dust and all other tested ions including Cl^-^, SO_4_^2-^, Na^+^, K^+^, Mg^2+^, and Ca^2+^ occurred in D49 (Supplementary Table S4). Variations in dust and ion concentrations are commonly found at different depths of an ice core (Thompson et al., 1997; Miteva et al., 2015) and they probably contribute to the differences in their microbial communities. For example, a study of microorganisms in a high Arctic glacier revealed sulfate-reducing bacteria from the basal ice-containing sulfate (Skidmore et al., 2000). Calcium concentrations positively correlated with bacterial abundance in an ice core retrieved from Mount Geladaindong on the Tibetan Plateau (Yao et al., 2008). Dust particle concentrations were reported to correlate with microbial concentrations in ice cores in many studies (e.g., Abyzov et al., 1998; Miteva et al., 2009; Segawa et al., 2010). Our results suggest that the differences in the microbial communities between samples D41 and D49 probably reflect the difference in the concentrations of dust and many ions in these samples, and that the GS3 ice core contains valuable information about changes in microbial communities over the past ∼30,000 years.

### Microbial Community Clusters by Glacier

**FIGURE 5:**
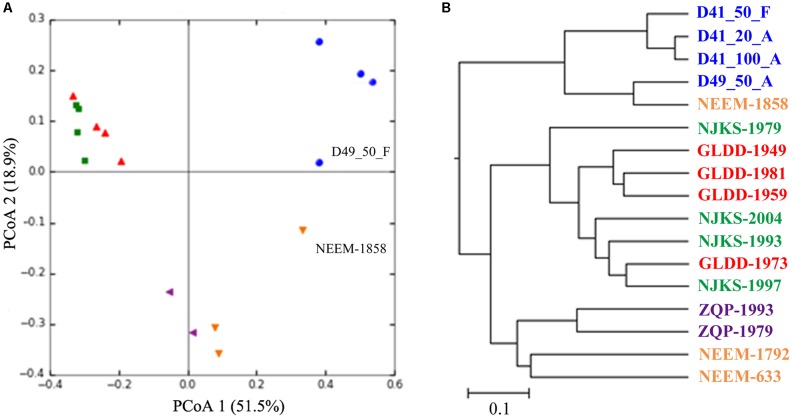
Relationships between individual samples illustrated by PCoA plots **(A)** and UniFrac tree (UPGMA, **B**). Both analyses were performed on the basis of the weighted UniFrac metric. Symbols of the same color indicate samples from the same glacier/ice core: blue color, GS3; red, Geladangdong (GLDD) Glacier; green, Noijinkangsang (NJKS) Glacier; purple, Zuoqiupu (ZQP) Glacier; orange, North Greenland Eemian Ice Drilling (NEEM) ice core.

As noted above the Guliya ice cap is the highest, largest, thickest, and coldest ice cap among all the ice caps in middle–low latitude regions (Yao et al., 1992; Thompson et al., 1995). Considering the distinct characteristics of the Guliya ice cap, we next compared the microbial communities of the Guliya ice cap samples with those from four other glaciers. These samples were chosen because the microbial communities of all these glaciers were investigated with the overlapped region (V4) of 16S rRNA genes using a next-generation sequencing strategy. PCoA of the microbial communities of Guliya and four other ice cap samples indicated that the communities varied among the glaciers and that the communities could be clustered by the ice cap (**Figure 5A**). The first and second dimensions of PCoA showed that the distribution of all samples accounted for 51.5 and 18.9% of community variability, respectively. The weighted UniFrac tree (UPGMA) also showed that most of the samples from a given ice cap formed a lineage (**Figure 5B**). Samples from GLDD and NJKS glaciers clustered together, indicating a closer relationship of their microbial communities. This finding agrees with the original report and might be attributed to the fact that both NJKS and GLDD glaciers are strongly influenced by the same westerly jet stream (Liu et al., 2016). Samples from the Guliya ice cap formed a separate and distant cluster outside the other samples, indicating that the Guliya ice cap might contain more distinct microbial community relative to the other glaciers in the Tibetan Plateau and Greenland. Interestingly, sample NEEM-1858 clustered with sample D49_50_F from the Guliya ice cap but not with other NEEM samples from Greenland (**Figure 5B**). This result can be attributed to the fact that both samples were dominated by the genus *Sphingomonas* with a relative abundance of 94.4 and 75.5% for NEEM-1858 and D49, respectively (data not shown).

The Guliya ice cap also shared some bacterial groups with other more distant glaciers, which supports the perspective that microorganisms are distributed everywhere in the world (Baas Becking, 1934). The two-tailed Mantel test indicated that microbial community compositions correlated significantly (*p* = 0.04) with the age of ice samples, suggesting that the variation in microbial community composition among these ice samples probably reflects unique climate conditions at the time they were deposited. Unfortunately, the relationships between microbial community composition and environmental parameters were not investigated in this study, because of the absence of relevant data in the other studies. In addition, the other ice samples were collected and analyzed in three different projects and laboratories. Although the microbial communities of all samples were analyzed with the overlapped gene region using a next-generation sequencing strategy, the difference in other experimental steps and/or methods (e.g., ice core drilling, DNA isolation, and investigators) likely also influences the microbial communities reported. Conflicting results of microbial content were reported in several papers investigating microorganisms in glacier ice (Willerslev et al., 2004). For example, the genus *Aquabacterium* was detected using 16S rRNA gene amplification and sequencing in the Lake Vostok ice samples (Christner et al., 2001), However, *Aquabacterium* was considered to be a contaminant because it was present in both the Lake Vostok ice sample and its negative control from another study (Priscu et al., 1999). As methodologies improve and cooperation increases among research groups around the world, it will be easier to compare ice core microbial communities generated from different laboratories and better understand the ecological implications of the ice microbial communities.

## Conclusion

Microbial communities in glacier ice with low biomass have been studied previously. However, as a laboratory new to this science, we sought to establish robust “background” controls and *in silico* “contaminant” removal protocols for our work with low-biomass ice samples. Our effort expands prior work that established *in silico* contaminant removal procedures (Ram, 2009; Knowlton et al., 2013; Miteva et al., 2015; Cameron et al., 2016); however, our study also expands the number of control samples (i.e., a co-processed sterile water artificial ice core, air samples collected from the ice processing laboratories, and a blank, sterile water sample) to generate “clean” datasets for further analysis. We used this method to investigate the microbial communities in ice from two depths in a GS3 ice core and found that significantly different microbial profiles were archived. Unfortunately, glaciers around the world are rapidly shrinking primarily due to the warming of the atmosphere in response to increasing concentrations of greenhouse gases released during the burning of fossil fuels (Zemp et al., 2015; Burkhart et al., 2017). This will lead to a gradual loss of the microbial information archived in glacier ice from which past climate and environmental changes may be reconstructed. The “clean” protocol procedures introduced in this study can now be used to help investigate low-biomass microbial communities preserved in Earth’s glaciers and ice caps. In addition, with further advancement of methods and technologies, such as metagenomics (Petrosino et al., 2009) and single-cell sequencing (Lasken, 2012), we will be able to better address microbial ecological questions for low-biomass, cold glacier ice, and bring microbial profiles into predictive ecological models of past climate changes in “frozen archive” environments.

## Author Contributions

Z-PZ, NS, MG, DK, EM-T, VR, JVE, LT, and MS conceived and designed the research, analyzed the data, and critically reviewed the manuscript. Z-PZ, NS, MG, and DK performed the laboratory experiments. Z-PZ, EM-T, VR, JVE, LT, and MS wrote the manuscript.

## Conflict of Interest Statement

The authors declare that the research was conducted in the absence of any commercial or financial relationships that could be construed as a potential conflict of interest.

## Footnotes

1 https://www.ncbi.nlm.nih.gov/books/NBK158900/
