# Supplementary material for: Clean Low-Biomass Procedures and Their Application to Ancient Ice Core Microorganisms
Source: Front Microbiol. 2018 May 25;9:1094. doi: 10.3389/fmicb.2018.01094 (PMC5992382; doi:10.3389/fmicb.2018.01094)
Supplement: Supplementary file 1 [file Data_Sheet_1.PDF]

Supplementary Material

Clean Low-biomass Procedures and Their Application to Ancient Ice Core Microorganisms

Zhi-Ping Zhong, Natalie E. Solonenko, Maria Consuelo Gazitúa, Donald V. Kenny, Ellen Mosley-Thompson, Virginia I. Rich, James L. Van Etten, Lonnie G. Thompson*, Matthew B. Sullivan*

***Correspondence:**

Lonnie G. Thompson: thompson.3@osu.edu; Matthew B. Sullivan: sullivan.948@osu.edu

Supplementary Figures and Tables

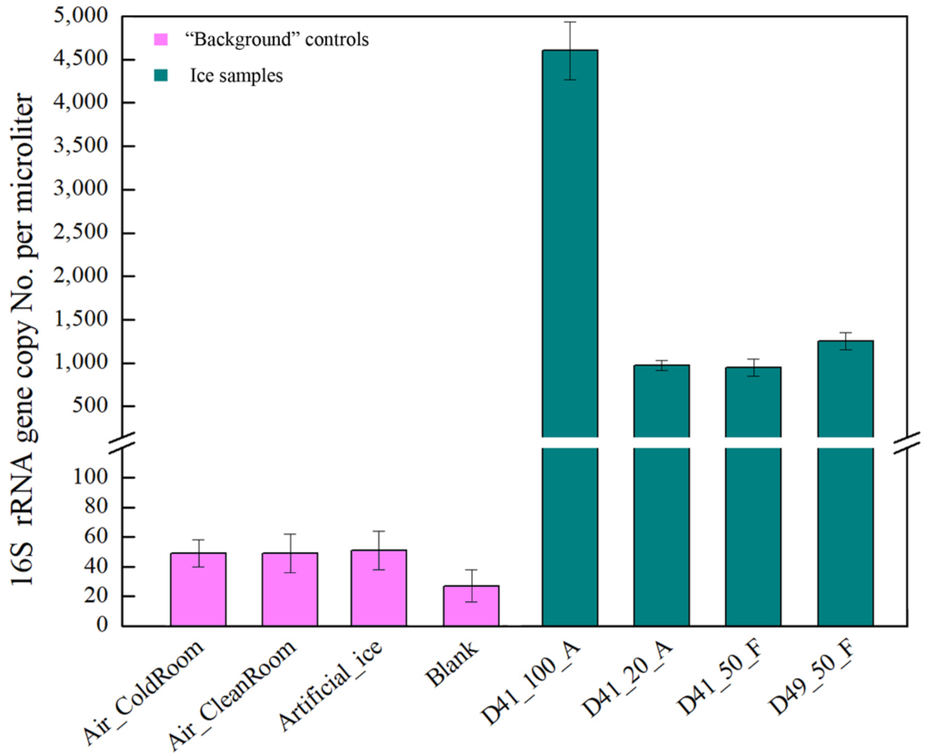

Supplementary Figure S1. Concentrations of microbial 16S rRNA genes in the 'background' controls and ice samples quantified by qPCR. Concentrations were calculated to 16S rRNA gene copy number per µl of the 50-µl elution volume for each sample. The four ice samples are described in Fig. 1. The 'background' controls: Blank, a blank sample with 400-ml sterile water; Artificial_ice, an artificial ice sample made by sterile water and processed along with the glacier ice samples; Air_ColdRoom and Air_CleanRoom, 2 air samples collected from a cold and clean room, respectively, in which the ice samples were processed. Bars represent SD.

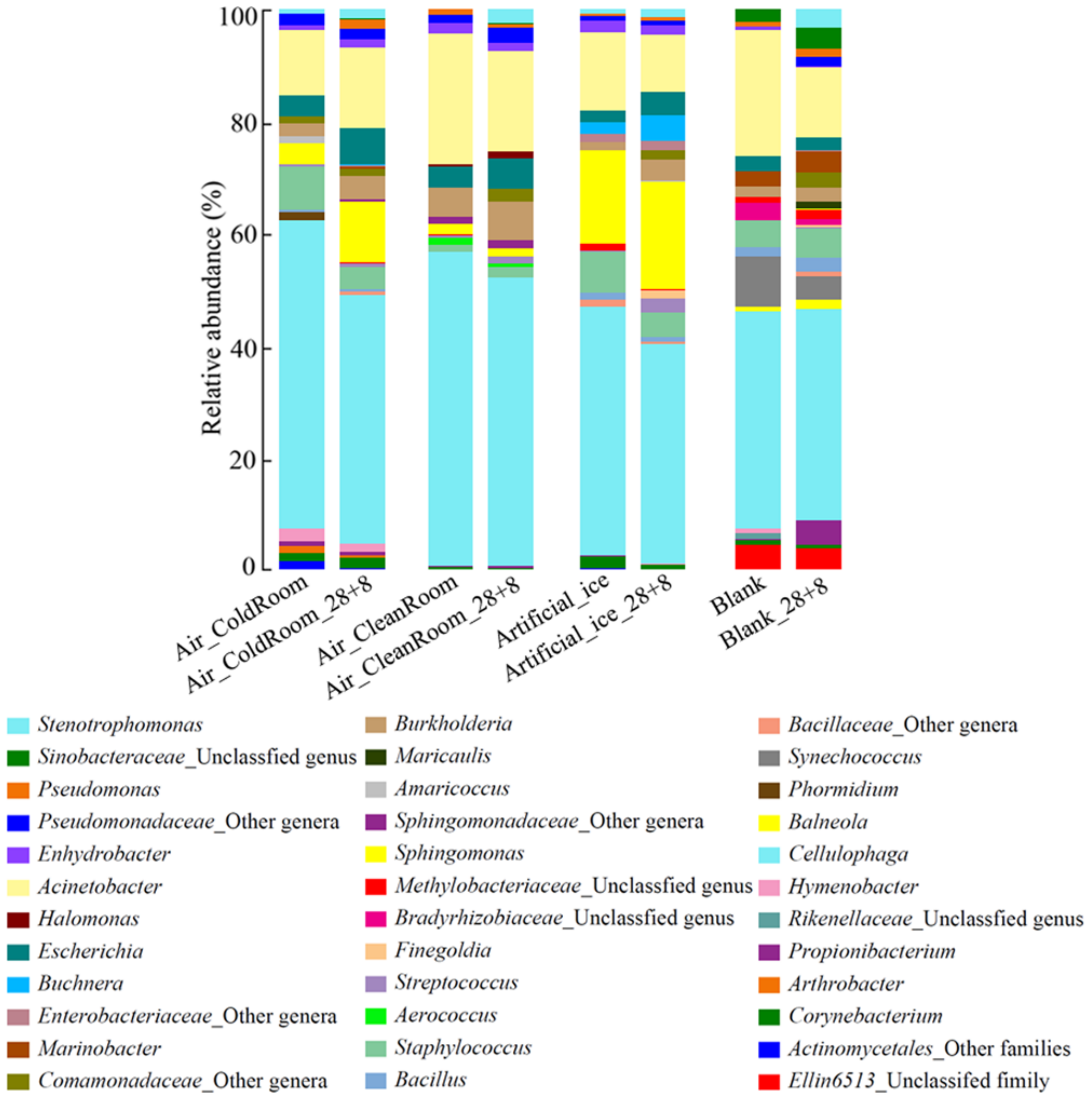

Supplementary Figure S2. Microbial community structure of the 4 pairs of original and pre-amplified ‘background’ controls. Only the 36 most abundant genera (relative abundance >1.0% in at least one sample) are included. The names of pre-amplified samples are coded as follows for the example of Air_ColdRoom_28+8: Air_ColdRoom, the original sample name as described in Fig. 1; 28+8, the cycle times of the first round PCR (28) and the reconditioning PCR (28), respectively. The ‘Other genera/families’ represent unclassified sequences and could not be assigned to a single genus/family.

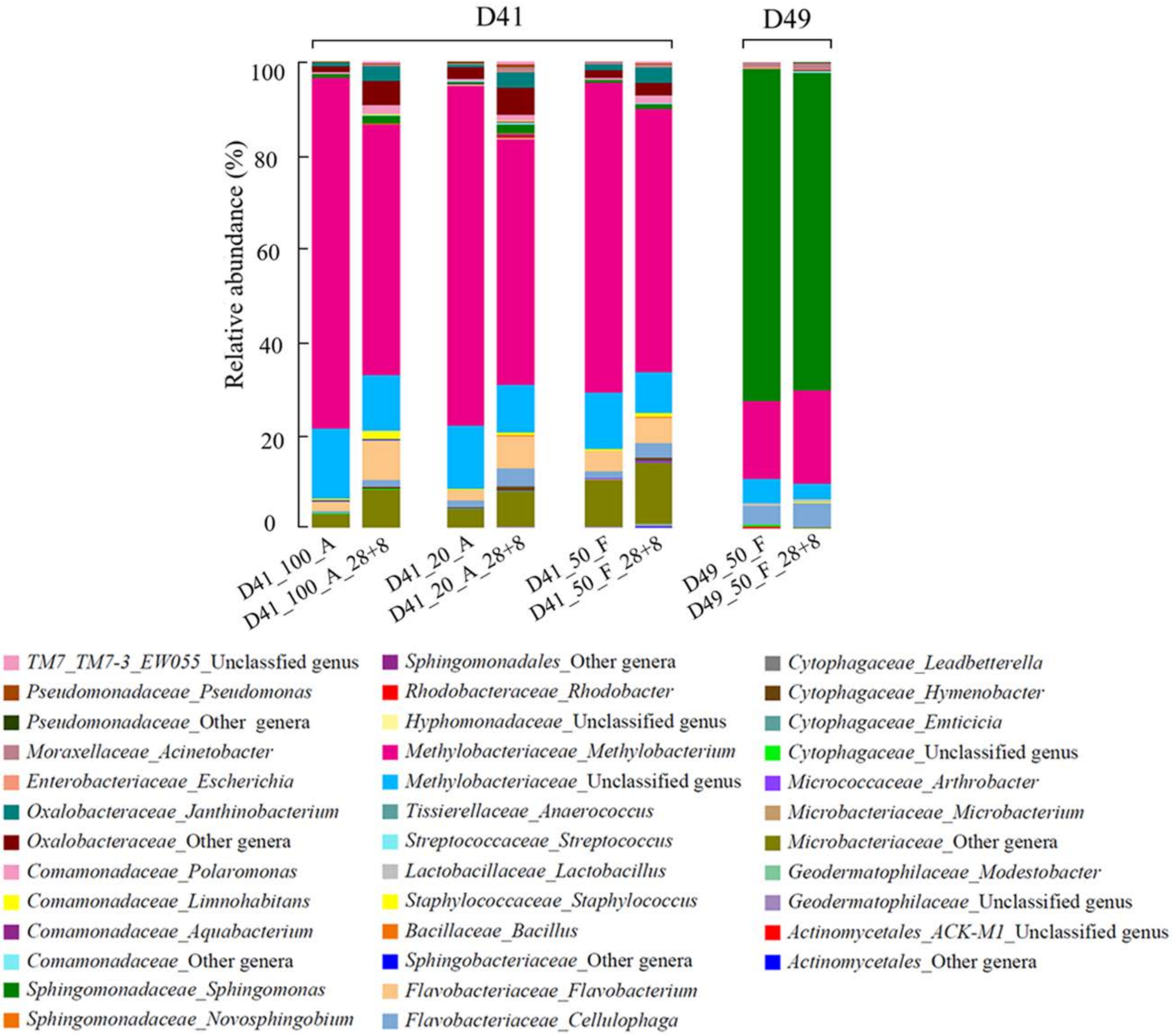

Supplementary Figure S3. Microbial community structure of the 4 pairs of original and pre-amplified GS3 ice core samples before *in silico* decontamination. All eight libraries were normalized to 10,400 sequences. Only the 37 most abundant genera (relative abundance >0.1% in at least one sample) are included. The names of pre-amplified samples are coded as described in Figure S2. The 'Other genera' represent unclassified sequences and could not be assigned to a single genus.

Supplementary Table S1 (Data Sheet 2.XLSX). Microbial community structure of the 4 'background' controls illustrated as relative abundance (%) of each genus

Supplementary Table S2 (Data Sheet 3.XLSX) Microbial community structure of the 4 pairs of original and pre-amplified 'background' controls illustrated as relative abundance (%) of the 36 most abundant genera

Supplementary Table S3 (Data Sheet 4.XLSX). Microbial community structure of the GS3 glacier ice samples illustrated as relative abundance (%) of each genus

Supplementary Table S4 (Data Sheet 5.XLSX). Physical and chemical characteristics of ice samples from the GS3 ice core
